# Down-Klinefelter Syndrome With Concurrent Double Aneuploidy in an Indian Child

**DOI:** 10.7759/cureus.55847

**Published:** 2024-03-09

**Authors:** Pradeep Kumar Gunasekaran, Pooja Jindal, Tanuja Rajial, Varuna Vyas, Kuldeep Singh

**Affiliations:** 1 Pediatrics, All India Institute of Medical Sciences, Jodhpur, Jodhpur, IND

**Keywords:** children, indian, concurrent aneuploidy, double aneuploidy, down-klinefelter syndrome

## Abstract

The genetics of Down syndrome (DS) and Klinefelter syndrome (KS) are a nondisjunction of autosomal and sex chromosomes, respectively, resulting in aneuploidies. Less than 70 cases of concurrent Down-Klinefelter syndrome (DS-KS) have been reported in the literature. We report the case of a five-month-old Indian child with a rare double aneuploidy resulting in DS-KS. A five-month-old boy born to non-consanguineously married parents presented with failure to thrive and dysmorphic facies. The family history was unremarkable. On examination, he had an upward eye slant, a depressed nasal bridge, a horizontal crease in the left hand, and a sandal gap. A clinical diagnosis of the Down phenotype was considered. Karyotype analysis revealed the presence of double aneuploidy (48, XXY,+21) suggestive of DS-KS. Down-Klinefelter syndrome presents with the DS phenotype at birth, and the characteristic KS phenotype develops in early infancy and apparently manifests during puberty only. Early diagnosis is required for parental counseling and planning for future pregnancies. In children with a typical Down syndrome phenotype, chromosomal analysis is highly recommended. The diagnosis of DS-KS at the earliest has implications for these children’s short-term and long-term outcomes. It helps in planning the subsequent pregnancy with appropriate genetic testing and counseling to avoid the risk of another child with trisomy.

## Introduction

The genetics of Down syndrome (DS) and Klinefelter syndrome (KS) are a nondisjunction of autosomal and sex chromosomes, respectively, resulting in aneuploidies. Down syndrome is one of the most common genetic causes of intellectual disability, more commonly due to trisomy on chromosome 21 [1]. Klinefelter syndrome presents with a broad clinical spectrum in a phenotypic male due to the presence of extra X chromosomes [2]. Less than 70 cases of concurrent Down-Klinefelter syndrome (DS-KS) have been reported in the literature. We report the case of a five-month-old Indian child with a rare double aneuploidy resulting in DS-KS.

## Case presentation

Chief complaints

A five-month-old boy presented to the pediatric OPD with failure to thrive and dysmorphic facies.

Perinatal history

The patient was first born to non-consanguineously married parents. He was born at 34+2 weeks of gestation, with a birth weight of 1.34 kg, and was admitted to the neonatal intensive care unit (NICU) for 20 days because of prematurity.

Family history

The family history of the patient was unremarkable.

Physical examination

**Figure 1:**
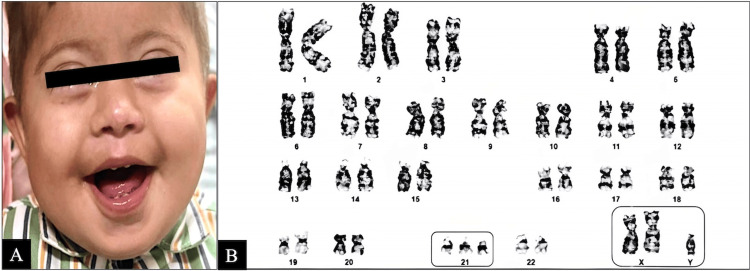
Down-Klinefelter syndrome and karyotype 48,XXY,+21 Figure 1A: Index child with Down phenotype; Figure 1B: Karyotyping analysis revealed 45 autosomes and three sex chromosomes with the presence of double aneuploidy: extra sex chromosome X (XXY pattern) and extra chromosome 21, consistent (marked in boxes) with Down-Klinefelter Syndrome (48,XXY,+21).

On examination, his weight was 2.8 kg (-7.2 Z-score), his length was 52 cm (-7.26 Z-score), and his head circumference was 34.6 cm (-7.13 Z-score). He had a wide-open anterior fontanelle, a prominent forehead, a triangular chin, an upward (mongoloid) eye slant, a depressed nasal bridge, a horizontal crease in the left hand, and a sandal gap suggestive of the Down phenotype (Figure 1A). He had a 2/6 grade systolic murmur on the cardiovascular examination, and the rest of the systemic examination was unremarkable. The ophthalmological examination was normal. Further relevant workup was done.

Laboratory examinations

The patient's hemogram, liver and renal function tests, and thyroid profile were within normal limits.

Imaging examinations

The ultrasound (USG) of the abdomen and pelvis was normal. Two-dimensional (2D) echocardiography revealed a 2 mm ostium secundum atrial septal defect (ASD).

Genetic testing

Karyotype analysis revealed 45 autosomes and three sex chromosomes with double aneuploidy, including extra sex chromosome “X” (XXY pattern) and extra chromosome 21, consistent with DS-KS (Figure 1B). The final diagnosis based on the karyotyping was DS-KS.

Outcome and follow-up

At present, he is being monitored on an outpatient basis by a multidisciplinary team.

## Discussion

Down syndrome (MIM#190685), the most common genetic cause of intellectual disability due to chromosomal aberrations (trisomy 21), was first described by John Langdon Haydon Down, an English physician, in 1862 [1]. The incidence of DS increases with maternal age and is estimated at 1:319 to 1:1000 live births, differing between populations [1]. The aneuploidy in DS is the presence of an extra copy of chromosome 21 due to meiotic nondisjunction (maternal in 90%), Robertsonian translocation (2%-4%), most commonly t (14;21), ring chromosome, or isochromosome. The karyotype in DS is 47,5 XY, +21 in males and 47, XX, +21 in females. The clinical phenotype of DS includes a distinct small face, mongoloid eye slant, hypertelorism, flat nasal bridge, small mouth, large tongue, single palmar crease, sandal toe gap, hypotonia, endocrinological disorders, mainly thyroid dysfunction, endocardial cushion defects, gastrointestinal tract anomalies, hematological malignancies, and learning disabilities in early infancy [1].

Klinefelter syndrome is the most common sex chromosome abnormality and primary hypogonadism, first described by Harry Fitch Klinefelter, an American endocrinologist, in 1942 [2]. The prevalence of KS is estimated at 1:500 to 1:1000 live male births, and the risk increases with both paternal and maternal age [3]. Aneuploidy in classic KS is the presence of one or more X chromosomes due to meiotic nondisjunction (maternal in 50%). The karyotype in KS is 47, XXY in 90%, mosaic type 47, XXY/46, and XY in 10% of patients [4]. The clinical phenotype of KS is a spectrum of manifestations, including micropenis, cryptorchidism, hypospadias, hypotonia, and developmental delay in infants. In childhood, the manifestations are small testes, relatively tall stature, hypertelorism, high-arched palate, gynecomastia, hypotonia, developmental delay predominantly in the language domain, and behavioral issues [2]. Learning disabilities occur in about 70% of KS patients. Less than 10% of boys with KS are diagnosed before puberty, and that too with the most severe phenotype [2]. Karyotyping analysis is used for confirmation of the KS diagnosis.

The first case of DS-KS with double aneuploidy (48, XXY, +21) was first described by Charles Edmund Ford, an English cytogeneticist, in 1959 [5]. The incidence of DS-KS is reported at 0.4 to 0.9 per 10,000 male births [6]. Studies found that the coincidence rate of DS-KS is 0.098%, approximately in the same individual [7]. The karyotype in double aneuploidy (DS-KS) is 48, XXY, +21, and the nondisjunction can be entirely maternal, entirely paternal, or both maternal and paternal in origin [6]. Kovaleva et al. found that increasing age was a significant risk factor for DS-KS double aneuploidy, with mean maternal and paternal ages of 33 and 38 years, respectively [7].

Also, it has been well established that there is an increased risk of subsequent births with aneuploidies after the birth of one child with trisomy. The clinical phenotype of DS-KS includes a usual presentation with the DS phenotype at birth, and the characteristic KS phenotype begins developing in early infancy. Only during puberty do these manifestations become apparent [6]. Based on a recent literature review by Alallah et al., only about 67 cases of DS-KS have been reported worldwide [8]. Among the reported cases, only 12 cases of DS-KS had congenital heart diseases, and ASD, ventricular septal defect, and patent ductus arteriosus were the most commonly reported defects [8]. Al Motawa et al. reported the first case of DS-KS with type 1 diabetes mellitus (T1DM) at 18 months of age [9].

The alterations resulting from the multiple aneuploidies may weaken, enhance, or not have modifying effects on each other [10]. Early diagnosis is required for parental counseling and planning for future pregnancies. Multimodal management is aimed at improving the overall quality of life for these patients, including specific treatment for congenital heart defects if present, speech therapy for language delay, and testosterone therapy at puberty for the development of secondary sexual characteristics to improve mood, behavior, and bone and muscle mass.

## Conclusions

In children with a typical Down syndrome phenotype, chromosomal analysis is highly recommended. Early diagnosis of DS-KS has implications for these children's short and long-term outcomes. It aids in the planning of subsequent pregnancies by providing appropriate genetic testing and counseling to reduce the risk of having another child with trisomy.

## References

[1] Asim A, Kumar A, Muthuswamy S, Jain S, Agarwal S (2015). "Down syndrome: an insight of the disease". J Biomed Sci.

[2] Bonomi M, Rochira V, Pasquali D, Balercia G, Jannini EA, Ferlin A (2017). Klinefelter syndrome (KS): genetics, clinical phenotype and hypogonadism. J Endocrinol Invest.

[3] Bojesen A, Juul S, Gravholt CH (2003). Prenatal and postnatal prevalence of Klinefelter syndrome: a national registry study. J Clin Endocrinol Metab.

[4] Gravholt CH, Chang S, Wallentin M, Fedder J, Moore P, Skakkebæk A (2018). Klinefelter syndrome: integrating genetics, neuropsychology, and endocrinology. Endocr Rev.

[5] Ford CE, Jones KW, Miller OJ, Mittwoch U, Penrose LS, Ridler M, Shapiro A (1959). The chromosomes in a patient showing both mongolism and the Klinefelter syndrome. Lancet.

[6] Jeanty C, Turner C (2009). Prenatal diagnosis of double aneuploidy, 48,XXY,+21, and review of the literature. J Ultrasound Med.

[7] Kovaleva NV, Mutton DE (2005). Epidemiology of double aneuploidies involving chromosome 21 and the sex chromosomes. Am J Med Genet A.

[8] Alallah J, Habhab S, Mohtisham F, Shawli A, Daghistani M (2022). Down-Klinefelter syndrome (48,XXY,+21) in a Saudi neonate: a case report and literature review. Cureus.

[9] Al Motawa MN, Al Buali MJ, Al Agnam AA, Alibraheem AA, Bu Zaid HT (2022). Rare double aneuploidy (Down-Klinefelter syndrome): a case report. Cureus.

[10] Pinti E, Lengyel A, Fekete G, Haltrich I (2020). What should we consider in the case of combined Down- and 47,XY,+i(X)(q10) Klinefelter syndromes? The unique case of a male newborn and review of the literature. BMC Pediatr.

